# Supplementary material for: Stakeholder Engagement in Developing an Electronic Clinical Support Tool for Tobacco Prevention in Adolescent Primary Care
Source: Children (Basel). 2018 Dec 17;5(12):170. doi: 10.3390/children5120170 (PMC6306818; doi:10.3390/children5120170)
Supplement: Supplementary file 1 [file children-05-00170-s001.pdf]

Supplementary File 1

UF Health Pediatrics Tobacco Survey*

Have you ever used any of the following tobacco products, even one or two times? Check all that apply.

- Cigarettes
- E-cigarettes or vaping products
- Cigars, cigarillos, or filtered cigars
- Hookah, shisha, or waterpipe
- Smokeless tobacco, such as snus pouches, snuff, dip or chewing tobacco
- I have never used any of the tobacco products listed above

Do you currently use any of the following tobacco products, every day or some days? Check all that apply.

- Cigarettes
- E-cigarettes or vaping products
- Cigars, cigarillos, or filtered cigars
- Hookah, shisha, or waterpipe
- Smokeless tobacco, such as snus pouches, snuff, dip or chewing tobacco

If one of your friends offered you a cigarette, e-cigarette, or another tobacco product, would you use it?

- Definitely yes
- Probably yes
- Probably not
- Definitely not
- Don't know

At any time during the next 12 months, do you think you will use cigarettes, e-cigarettes, or another tobacco product?

- Definitely yes
- Probably yes
- Probably not
- Definitely not
- Don't know

Do you think you will be using cigarettes, e-cigarettes, or another tobacco product 5 years from now?

- Definitely yes
- Probably yes
- Probably not
- Definitely not
- Don't know

About how long has it been since you last smoked a cigarette, or used an e-cigarette or any other tobacco product?

- less than 24 hours
- from 1 to 7 days
- from 8 to 30 days
- from 31 to 90 days
- more than 90 days
- I have never smoked cigarettes or used e-cigarettes or other tobacco products

Does anyone who lives with you now use any of the following tobacco products? Check all that apply

- Cigarettes
- E-cigarettes or vaping products
- Cigars, cigarillos, or filtered cigars
- Hookah, Shisha, or waterpipe
- Smokeless tobacco, such as snus pouches, loose snus, moist snuff, dip, spit or chewing tobacco

Considering the following statements, which one is the most Important for you to discuss with your doctor?
I am worried that if I use tobacco...

- I will be exposed to harmful chemicals.
- I will have gross looking teeth.
- I will have gross looking skin.
- I will be unpopular.
- I will develop a serious disease.
- I will waste money I could have spent on other things.
- I will be hooked.

Please view the video below and then click the "continue" button.

[VIDEO – participant views matched videos based on response to preceding ranking question and tobacco product(s) used]

Please rate this video.

- Like
- Dislike

Please review your responses. To finish, click Submit Questionnaire. Or, click any question to modify an answer.

* The tobacco survey is combined with the American Academy of Pediatrics Bright Futures health risk assessment. To view the Bright Futures instruments (varies by patient age), visit: https://brightfutures.aap.org/
